# Trust me if you can: a survey on reliability and interpretability of machine learning approaches for drug sensitivity prediction in cancer

**DOI:** 10.1093/bib/bbae379

**Published:** 2024-08-05

**Authors:** Kerstin Lenhof, Lea Eckhart, Lisa-Marie Rolli, Hans-Peter Lenhof

**Affiliations:** Center for Bioinformatics, Chair for Bioinformatics, Saarland Informatics Campus (E2.1) Saarland University, Campus, D-66123 Saarbrücken, Saarland, Germany; Center for Bioinformatics, Chair for Bioinformatics, Saarland Informatics Campus (E2.1) Saarland University, Campus, D-66123 Saarbrücken, Saarland, Germany; Center for Bioinformatics, Chair for Bioinformatics, Saarland Informatics Campus (E2.1) Saarland University, Campus, D-66123 Saarbrücken, Saarland, Germany; Center for Bioinformatics, Chair for Bioinformatics, Saarland Informatics Campus (E2.1) Saarland University, Campus, D-66123 Saarbrücken, Saarland, Germany

**Keywords:** trustworthiness, reliability, interpretability, anti-cancer drug sensitivity prediction, uncertainty

## Abstract

With the ever-increasing number of artificial intelligence (AI) systems, mitigating risks associated with their use has become one of the most urgent scientific and societal issues. To this end, the European Union passed the EU AI Act, proposing solution strategies that can be summarized under the umbrella term trustworthiness. In anti-cancer drug sensitivity prediction, machine learning (ML) methods are developed for application in medical decision support systems, which require an extraordinary level of trustworthiness. This review offers an overview of the ML landscape of methods for anti-cancer drug sensitivity prediction, including a brief introduction to the four major ML realms (supervised, unsupervised, semi-supervised, and reinforcement learning). In particular, we address the question to what extent trustworthiness-related properties, more specifically, interpretability and reliability, have been incorporated into anti-cancer drug sensitivity prediction methods over the previous decade. In total, we analyzed 36 papers with approaches for anti-cancer drug sensitivity prediction. Our results indicate that the need for reliability has hardly been addressed so far. Interpretability, on the other hand, has often been considered for model development. However, the concept is rather used intuitively, lacking clear definitions. Thus, we propose an easily extensible taxonomy for interpretability, unifying all prevalent connotations explicitly or implicitly used within the field.

## Introduction

While the birth of artificial intelligence (AI) as an academic discipline dates back to the 1950s, after decades of research and development, AI-based systems have become omnipresent: In 2022, roughly 35$\%$ of companies have been integrating AI-based software in their workflows [1]. In fact, AI-based systems have already arrived in our everyday lives. Social media platforms like Instagram and streaming services like Netflix use machine learning (ML) based recommendation algorithms [2, 3], search engines such as Google use AI to improve the search [4] to provide the most relevant search results for the specific user, and automobile manufacturers use AI systems for self-driving cars [5]. It is anticipated that AI-based systems will augment our lives tremendously. In particular, the healthcare sector is also conducting extensive research into AI-based strategies to improve the diagnosis, prognosis, and therapy of diseases. However, the unmonitored and unrestricted use of AI systems may jeopardize the benefits they were planned for.

Therefore, the European Commission formed an expert group that, in 2019, published guidelines for implementing trustworthy AI approaches [6]. Here, trustworthiness is defined as a set of properties that should guarantee lawful, ethical, and robust use of AI systems in practice [6]. Especially for high-stake application cases such as typically encountered in medicine, we need to demand the highest degree of compliance with these guidelines.

In medicine, the development of decision support systems for diagnostic, prognostic, or therapeutic purposes has long been a focus of attention [7–12]. In general, the idea of these systems is to match patient data to some form of pre-existing knowledge to derive patient-specific recommendations that serve as guidance for physicians. During the previous decade, there have been an increasing number of research efforts to boost the usefulness of decision support systems with AI algorithms, especially in the context of complex diseases such as cancer [7]. In cancer research specifically, one main goal is to optimize (targeted) drug therapy using an AI-based interpretation of molecular patient data. In fact, there is a whole research area in bioinformatics that has been working on the putative design of AI tools, which are able to predict the effectiveness of anti-cancer drug treatment, for over a decade: drug sensitivity and synergy prediction. In this research field, ML models are developed to predict therapy responsiveness based on molecular data of cells from model systems (e.g. patient-derived xenografts and cell lines), often combined with chemical information of drugs. Moreover, the models are used to elucidate the relationship between the molecular characteristics of cells and therapy responsiveness [13]. While a plethora of different approaches has already been suggested for this task and there also exist concrete implementation ideas for such tools in decision support systems (e.g. [14]), there are no reports on the widespread real-world application as decision support tools yet. One main reason for this is data-related challenges [13, 15]: Since it is neither ethically justifiable nor technically feasible to explore the space of all possible treatments exhaustively in real clinical settings, ML models are trained on biomedical model system data (e.g. from patient-derived xenografts and cell lines) that are significantly more abundant than patient data but reflect tumor biology only to some extent. [16–18]. Although model system data are more abundant than patient data, this quantity is still comparatively small given their high dimensionality ($\sim $1000 cell lines versus tens of thousands of multi-omics measurements), impeding ML model training. Especially for samples of treatment success (drug-sensitive samples), the data are extremely scarce [19–21], leading to poor prediction performance. Such challenges hinder a straightforward translation of model system-derived results to patient data and create a lack of trust in predictions. However, with an appropriate design of ML methods, trust in their predictions could nevertheless be achieved. Moreover, independent of data-related issues, we have to demand the trustworthiness of these systems to ensure maximal benefit for patients while preventing any harm. For example, model decisions should be traceable, and uncertainties in the model should be reported. In this article, we address the question of to what extent trustworthiness has already been taken into account in the design of ML-based tools for anti-cancer drug treatment. In our analyses, we mainly focus on methods for monotherapy prediction, i.e. the prediction of a response from a cell line when treated with a single drug. However, we also included several well-known drug synergy prediction methods (predictions for synergistic effects of drug combinations) in our comparison [22–26].

Before we assess the trustworthiness of these methods, we need to discuss the properties that should guarantee trustworthiness. Traditionally, performance measures [e.g. mean squared error (MSE) for regression or Matthews correlation coefficient (MCC) for classification] that quantify the difference between a known true response and the model prediction have been used as the main quality metric in the ML model development more generally, and drug sensitivity prediction in particular. The evaluation of performance measures can serve as an indicator of performance for future predictions, thereby creating trust in a model. However, it is just a single building block in that respect:

(i) In a real application of our model, we wish to make predictions for new instances where no response is known. Consequently, we cannot evaluate a performance measure and would like to have a probability estimation for the correctness of the predictions of our model instead.
(ii) More generally speaking, performance evaluation does not justify or explain the decisions of the ML system.

To address the first problem, we would like to quantify the degree of trust we have in a prediction for a previously unseen instance, which we refer to as *reliability*. Given such a reliability estimate of a model, an expert may more easily decide to abstain from a proposed therapy of the ML model. The second mentioned problem can be solved by rendering models more interpretable. Intuitively speaking, *interpretability* is the extent to which a human can understand the decisions of a model. It may help a clinician to identify the molecular causes of therapy responsiveness. Thus, reliability and interpretability are particularly important properties of trustworthy AI systems.

In this article, we analyze to what extent these two aspects have already been taken into account in ML model development for drug sensitivity prediction. First of all, we explore what has been examined in other reviews in the area of drug sensitivity prediction.

In 2016, De Niz *et al*. [13] reviewed the field of drug sensitivity prediction, focusing on comparing the performance of four different ML approaches when applied to drug sensitivity data. They also discuss three different challenges when using these models as a decision support systems in actual clinical settings, i.e. data inconsistency, potential drug toxicology, and limited prediction accuracy. In the same year, Cortes-Ciriano *et al*. [27] revisited different ML approaches to predict drug sensitivity and discuss challenges in this field caused by the amount and quality of available data. In 2021, Sharifi-Noghabi *et al*. [15] published a review paper providing guidelines for developing ML models in drug sensitivity prediction. Their research should serve as a developers’ guidance in terms of datatype and dataset selection. Moreover, they report factors that can influence the model performance. In 2023, Partin *et al*. [28] dedicated themselves to reviewing the most popular method development direction of the current drug sensitivity prediction literature, i.e. deep learning methods. Their review focuses on methodological aspects of this field, in particular the diverse set of model architectures.

Even though trustworthiness is highly desirable in a medical application setting, none of the above-mentioned review articles explicitly addresses the reliability or interpretability of ML approaches in drug sensitivity prediction. In this article, we review the current drug sensitivity prediction landscape in terms of trustworthiness research with a focus on reliability and interpretability.

We reviewed 36 articles of the current drug sensitivity (31) and drug synergy (5) literature and, in the following text, discuss their work in terms of reliability and interpretability. In summary, we could reveal that hardly any reliability research has been carried out over the last decade: only two approaches take this concept into account [29, 30]. A different picture emerges for research in terms of interpretability: 22 articles are dedicated to this topic [19–21, 24, 25, 29–45]. However, although the term interpretability is intuitively understandable, there is no general definition for this term [28, 46, 47]. In particular, Partin *et al*. [28] also noted that for drug sensitivity prediction such a definition is absent but would be crucial to advance methodological developments for deep learning methods. Since no definition is given in the mentioned articles, this could lead to ambiguities and misconceptions. This also reflects the fact that the concept of interpretability has different connotations in the ML field [47]. To address this issue, we propose a general taxonomy for the term interpretability and categorize recent work on drug sensitivity accordingly. In this way, we can easily recognize which types of interpretability have hardly been explored so far. Moreover, this taxonomy may serve as a sound and extensible basis for the development and categorization of future interpretability methods.

We structured this review article as follows: First, we provide definitions for the four major ML realms, i.e. supervised, unsupervised, semi-supervised, and reinforcement learning. Here, we also place the corresponding papers from drug sensitivity prediction in these four realms. Next, we start our discussion on trustworthiness with performance. Then, we define reliability and assess the investigated papers in this respect. Afterwards, we explain the different notions of interpretability that are currently used and derive a taxonomy that captures their differences. Lastly, we discuss future research directions for trustworthy ML based on our findings. In particular, we elaborate on out-of-distribution estimation, which is an integral part of model reliability but has not been considered by any of the presented approaches.

## The four realms of ML

**Figure 1:**
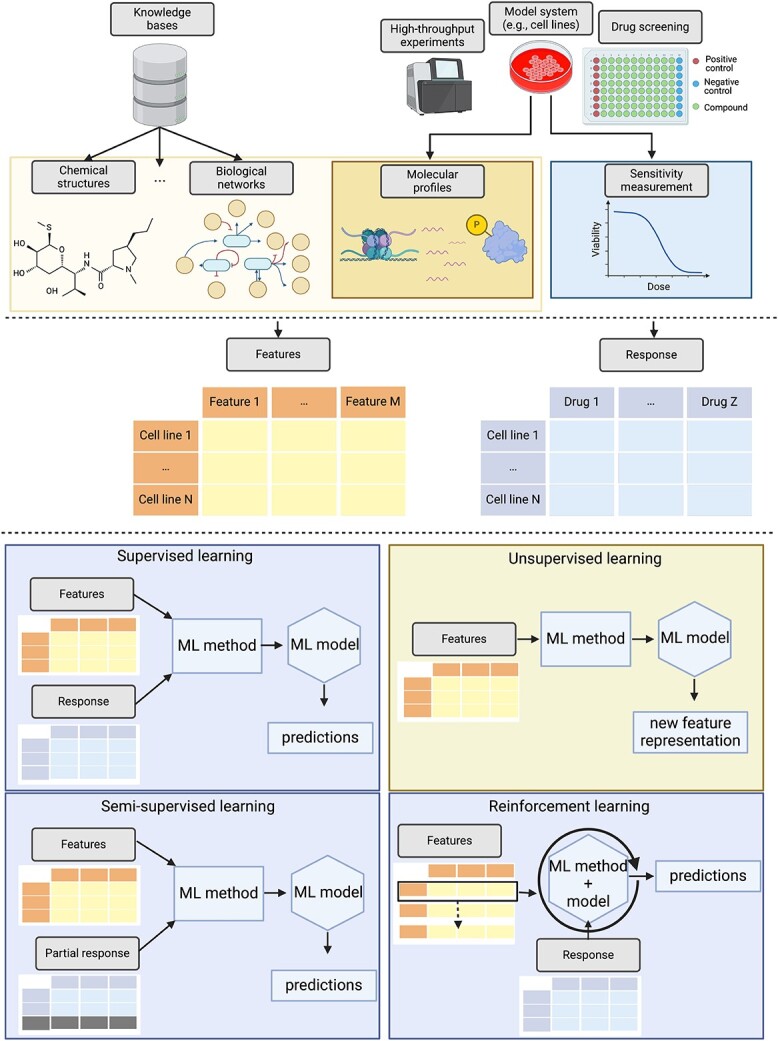
**Overview of ML workflows for drug sensitivity prediction in cancer;** in this figure, we depict typical ML workflows of the four different ML realms for drug sensitivity prediction in cancer; independent of the ML realm, the data stem from a variety of biological experiments; the primary data source of all drug sensitivity prediction approaches is usually generated as follows: model systems, e.g. cancer cell lines, are molecularly characterized and subject to high-throughput drug screening; this results in molecular features (e.g. mutations, copy number variations, and gene expression values) of the cell lines complemented with drug response values for a variety of drugs; these primary data are often combined with biomedical knowledge from a diverse range of knowledgebases to enhance the overall characterization of the model system, i.e. the features; supervised, semi-supervised, and reinforcement learning ultimately utilize both: the drug response and the feature data to generate a model able to predict drug responses for previously unseen data points; in contrast, unsupervised learning is generally applied to the feature space only to obtain a new feature representation; this new representation can then be employed in the former three ML realms; created with BioRender.com

ML is a multidisciplinary research area sharing its methodology with various other disciplines such as optimization, statistics, and information theory. Yet, what all ML methods have in common is that they are employed for learning from data. Typically, the ML landscape can be divided into four major realms, each designed to fulfill specific tasks: supervised learning, unsupervised learning, semi-supervised learning, and reinforcement learning [48]. In the following text, we briefly contrast the four different realms and place the corresponding papers from anti-cancer drug sensitivity prediction in these four realms. In Fig. 1, we depict typical data sources and ML workflows of all four realms for drug sensitivity prediction in cancer.

### Supervised learning

In supervised learning, we are given a set of observed pairs $\{(x_{1}, y_{1}), (x_{2}, y_{2}), \cdots , (x_{N}, y_{N})\}$, where $x_{i} \in \mathscr{X} $ is called the feature vector for a sample $i$ and $y_{i} \in \mathscr{Y}$ the respective response. We assume that there is an unknown functional relationship between $\mathscr{X} $ and $\mathscr{Y} $, i.e. $f: \mathscr{X} \rightarrow \mathscr{Y} $. Usually, we employ a matrix-vector notation to represent the observed pairs. Here, $\mathbf{X}$ is an $N\ \times\ M$-matrix where $x_{i}$ is a row vector containing values of $M$ features. The vector $\mathbf{y}$ is the accordingly ordered $N$-dimensional response vector with each entry corresponding to one $y_{i}$. Using $f$, we can express $y_{i}$ as follows:


(1) \begin{align*}& y_{i} = f(x_{i}) + \epsilon\end{align*}

Note that we introduced a random error term $\epsilon $ that represents the measurement noise. We assume $\epsilon $ to be independent of $\mathbf{X}$ with mean zero [49]. Most supervised ML algorithms additionally assume that the aforementioned pairs are drawn iid, which means that the samples are **i**ndependent of each other and drawn from an **i**dentical probability **d**istribution with range $ \mathscr{X} \times \mathscr{Y} $ [50]. However, especially the first assumption is frequently violated by real-world (biological) data.

The task is to find the model $\hat{f}$ that best approximates $f$ [50, 51]. Depending on whether the values of $\mathbf{y}$ are continuous or discrete, we call this task regression or classification. We hypothesize that $\hat{f}$ belongs to a specific model type (hypothesis space) $\mathscr{H}$ containing mappings $h: \mathscr{X} \rightarrow \mathscr{Y}$. We obtain $\hat{f}$ by minimizing over some loss function $l: \mathscr{Y} \times \mathscr{Y} $ within an empirical risk function $R_{emp}(h)$ [52], i.e.


(2) \begin{align*}& R_{\textrm{emp}}(h) = \frac{1}{N} \sum_{i=1}^{N} l(h(x_{i}),y_{i})\end{align*}

and


(3) \begin{align*}& \hat{f} = \mathrm{argmin}_{h} R_{\mathrm{emp}}(h).\end{align*}

By following this approach, our prediction carries a variety of uncertainties, which we will discuss in depth in Section 4 (Reliability).

The straightforward modeling of drug response prediction in terms of ML is via supervised learning and most publications fall within this realm (cf. Table 1). Here, the samples are cancer cell lines that are characterized by multi-omics measurements, e.g. gene expression values. Each feature vector consists of $M$ entries and each entry corresponds to the expression of one gene. The response is the observed drug sensitivity from a drug screening assay reported in the form of some summary metric such as IC50 or AUC [53]. Consequently, each row of the model matrix $\mathbf{X}$ then contains the characterization of one cell line, and each entry of the response vector $\mathbf{y}$ is the measured sensitivity. While drug response prediction is inherently a regression task, it can be formulated as a classification task by discretization of the continuous sensitivity value [19–21, 44, 45, 54–56].

**Table 1:** Arrangement of DS literature **Placement of drug sensitivity literature in ML;** this table characterizes 36 drug sensitivity prediction approaches in terms of interpretability, reliability, and ML realm; ML realms are divided into supervised learning, semi-supervised learning, and reinforcement learning. The supervised learning category is further sub-divided into regression, classification, and regression and/or classification. The last category contains methods that can jointly perform both tasks or were applied to both tasks.

		Interpretability								Reliability
		Transparency			Explainability					
		Simulatability	Decomposability	Algorithmic transparency	Feature	Sample	Counterfactual	Concept	Model	
Supervised (regression)	Menden *et al*. (2013) [61]	✗	✗	✗	✗	✗	✗	✗	✗	✗
	Zhang *et al*. (2015) [31]	✗	✓	✗	✗	✗	✗	✗	✗	✗
	SRMF (2017) [32]	✗	✓	✗	✗	✗	✗	✗	✗	✗
	HARF (2017) [33]	✗	✓	✗	✗	✗	✗	✗	✗	✗
	Matlock el al. (2018) [35]	✗	✓	✗	✗	✗	✗	✗	✗	✗
	TreeCombo (2018) [24]	✗	✓	✗	✓	✗	✗	✗	✗	✗
	KRL (2018) [59]	✗	✗	✗	✗	✗	✗	✗	✗	✗
	RWEN (2018) [36]	✗	✓	✗	✗	✗	✗	✗	✗	✗
	CDRscan (2018) [62]	✗	✗	✗	✗	✗	✗	✗	✗	✗
	QRF (2018) [29]	✗	✓	✗	✓	✗	✗	✗	✗	✓
	NCFGER (2018) [37]	✗	✓	✗	✗	✗	✗	✗	✗	✗
	DeepDR (2019) [58]	✗	✗	✗	✗	✗	✗	✗	✗	✗
	netBITE (2019) [38]	✗	✓	✗	✗	✗	✗	✗	✗	✗
	Deng *et al*. (2020) [39]	✗	✓	✗	✗	✗	✓	✗	✗	✗
	Ahmed *et al*. (2020) [40]	✗	✓	✗	✗	✗	✗	✗	✗	✗
	ADRML (2020) [41]	✗	✓	✗	✗	✗	✗	✗	✗	✗
	PathDSP (2021) [42]	✗	✗	✗	✓	✗	✗	✗	✗	✗
	REFINED CNN (2021) [63]	✗	✗	✗	✗	✗	✗	✗	✗	✗
	GraphDRP (2021) [43]	✗	✓	✗	✓	✗	✗	✗	✗	✗
	Precily (2022) [64]	✗	✗	✗	✗	✗	✗	✗	✗	✗
Supervised (regression and/or classification)	KBMTL (2014) [56]	✗	✗	✗	✗	✗	✗	✗	✗	✗
	DeepSynergy (2018) [22]	✗	✗	✗	✗	✗	✗	✗	✗	✗
	DeepCDR (2020) [44]	✗	✗	✗	✓	✗	✗	✗	✗	✗
	Kim *et al*. (2021) [23]	✗	✗	✗	✗	✗	✗	✗	✗	✗
	MatchMaker (2022) [26]	✗	✗	✗	✗	✗	✗	✗	✗	✗
	SAURON-RF (2022) [21]	✗	✓	✗	✓	✗	✗	✗	✗	✗
	reliable SAURON-RF (2023) [30]	✗	✓	✗	✗	✗	✗	✗	✗	✓
Supervised (classification)	LOBICO (2016) [19]	✓	✓	✓	✓	✗	✗	✗	✗	✗
	Stanfield *et al*. (2017) [45]	✗	✓	✗	✗	✗	✗	✗	✗	✗
	SyDRa (2017) [25]	✗	✓	✗	✗	✗	✗	✗	✗	✗
	HNMDRP (2018) [34]	✗	✓	✗	✗	✗	✗	✗	✗	✗
	pLETORg (2018) [55]	✗	✗	✗	✗	✗	✗	✗	✗	✗
	Deep-Resp-Forest (2019) [54]	✗	✗	✗	✗	✗	✗	✗	✗	✗
	MERIDA (2021) [20]	✓	✓	✓	✗	✗	✗	✗	✗	✗
Semi	Dr.VAE (2019) [60]	✗	✗	✗	✗	✗	✗	✗	✗	✗
Reinf.	PPORank (2022) [65]	✗	✗	✗	✗	✗	✗	✗	✗	✗

### Unsupervised learning

Unsupervised learning can be interpreted as the task of finding interesting structures in data without a specific variable that guides or supervises the model [50]. Thus, in contrast to supervised learning, we have the model matrix $\mathbf{X}$, generated by drawing samples iid from a distribution with range $\mathscr{X}$, but no designated response vector $\mathbf{y}$. In low-dimensional spaces ($M \ll N$), we usually estimate the density of the distribution. However, high-dimensionality often seems to necessitate the usage of simpler approaches that more loosely learn the structure of the data [57]. Examples of such approaches include principal component analysis (PCA), clustering, and association rule mining [57].

Drug response prediction is not modeled as unsupervised learning task (cf. Table 1). Yet, unsupervised learning methods can still fulfill various functions within the task of drug response prediction: Since data obtained from high-throughput multi-omics measurements suffer from the curse of dimensionality ($M \gg N$), unsupervised learning algorithms such as PCA or autoencoders can be employed for reducing the dimensionality of the design matrix before training the ML model [58–60]. Clustering algorithms, e.g. k-medoid clustering, can help to divide the samples into groups when a group association is not known beforehand [30].

### Semi-supervised learning

Conceptually, classical semi-supervised learning lies between supervised and unsupervised learning: the model matrix $\mathbf{X}$ can be divided into two parts, one sub-matrix $\mathbf{X}^{r}$ with associated response vector $\mathbf{y}^{r}$, and a second sub-matrix $\mathbf{X}^{w}$ without an associated response vector. One possible approach would be to train a supervised model on $\mathbf{X}^{r}$ and then apply the resulting predictor to $\mathbf{X}^{w}$, i.e. to interpret this setting as a supervised learning application. However, we could also argue that the unlabeled data provide additional information on the structure of our data space, and thus can improve our model. There exist three popular assumptions describing this idea, i.e. the smoothness assumption, the low density assumption, and the cluster assumption.

These three assumptions may even be interpreted as different phrasings of the same principle: if two points $x_{i}$ and $x_{j}$ from a high-density region lie close by, they should not be separated by a decision boundary, i.e. their class labels $y_{i}$ and $y_{j}$ should be equal. Currently, we are only aware of one approach that employs semi-supervised learning for drug sensitivity prediction: Dr.VAE by Rampášek et al. [60] employs both pre- and post-treatment gene expression data from cell lines to predict their drug response. However, neither the post-treatment gene expression data nor the drug responses are available for all cell lines. In the final model, cell lines without post-treatment expression and cell lines without drug response are integrated and influence predictions.

### Reinforcement learning

In contrast to the previously discussed types of ML, reinforcement learning involves learning dynamically from interactions with an environment rather than from a fixed set of data points [66]. Here, an agent takes actions from a set of possible actions, while an environment can be in a set of different states. Whenever the agent performs an action, the environment provides feedback in the form of a reward or penalty and changes its state [67]. The goal of the agent is to maximize its reward, making reinforcement learning similar to supervised learning since the environment provides some form of supervision. However, there are also unique challenges to reinforcement learning, such as the need to explore the environment, known as trial and error search, and the principle of delayed reward, i.e. the action an agent takes at a specific point in time may not only influence the direct reward but also all future rewards. In the context of drug response prediction, reinforcement learning has not been extensively explored yet (cf. Table 1). However, we can interpret drug prioritization as a reinforcement learning task. For example, PPORank proposed by Liu *et al*. [65] assumes that an agent needs to generate the correct (given) ranking of drugs. The agent constructs the ranking one drug at a time. Here, each time step corresponds to one ranking position ordered from the most efficient to the least efficient. Based on the difference in efficiency between the selected and the actual drug at the current position, the environment returns a reward or penalty.

## Performance

Clearly, an ML system is only helpful if its output is correct, i.e. if it learned to perform a specific task. In a typical ML workflow for supervised, semi-supervised, and reinforcement learning, we rely on the assessment of the model performance in terms of the difference between the known and the predicted response. Typically, we optimize for performance during model training and finally compare different models based on the performance on a dedicated test set unseen during training. For regression, the (root) MSE or the Pearson correlation are commonly used performance measures (e.g. [21, 30, 31, 33, 37]). For classification, accuracy, sensitivity, specificity, and MCC are often employed (e.g. [19–21, 54]). We refer the interested reader to Naser and Alavi [68] for definitions of the most prevalent performance metrics. Performance evaluation can already provide some indication of whether we can trust a prediction. In particular, performance evaluation on data that systematically deviate from the training data (e.g. data collected at different time points or from different sources), such as performed in robustness analysis can be beneficial [69, 70]. In drug sensitivity prediction, performance has been the main model quality criterion and most—if not all—papers conduct some form of performance comparison to demonstrate the capabilities of the presented approach. However, directly comparing the reported performances between papers is impeded by the use of different data sets, sensitivity measures, and performance metrics [15]. Therefore, several benchmarking studies have been conducted (cf. Table 2). Their findings indicate that given the current data situation,

(i) gene expression is the most predictive omics-type,
(ii) simple models are competitive to complex models (in particular, all sorts of deep neural networks), and
(iii) highly drug-sensitive samples (head of the drug response distribution) are relatively poorly predicted.

**Table 2:** **Drug sensitivity benchmarking studies;** this table provides an overview on benchmarking studies for anti-cancer drug sensitivity prediction. We summarize the study design and the key findings of the paper.

	Description	Key findings
Jang *et al*. (2014) [71]	$\bullet\ $ Seven conventional ML algorithms (principal component regression, partial least square regression, support vector machines, RFs, lasso, ridge regression, elastic net) $\bullet\ $ four omics-types (gene expression, mutations, copy number variations, tumor type) $\bullet\ $ seven response metrics (IC50, AUC, ActArea, EC50 and four binary discretizations) $\bullet\ $ two datasets (CCLE, GDSC)	$\bullet\ $ elastic net and ridge regression performed best $\bullet\ $ gene expression is the most predictive datatype
Chen *et al*. (2021) [72]	$\bullet\ $ 14 state-of-the-art ML methods $\bullet\ $ three conventional ML methods (elastic net, RF, ridge regression) $\bullet\ $ four datasets (GDSC, CCLE, NCI-60, CTRP) $\bullet\ $ nine performance measures	$\bullet\ $ matrix factorization performed best $\bullet\ $ gene expression in the most predictive datatype but the addition of drug structure data and protein interactions is beneficial $\bullet\ $ Head and tail of the drug response distributions are relatively poorly predicted
Chen *et al*. (2022) [73]	$\bullet\ $ Seven state-of-the-art ML methods (mostly deep learning), elastic net $\bullet\ $ performance evaluation for previously unseen cell lines and drugs	$\bullet\ $ deep learning models are prone to overfitting and their performance deteriorates in drug-blind tests
Li *et al*. (2023) [74]	$\bullet\ $ Four state-of-the-art pathway-based deep learning methods $\bullet\ $ two conventional ML methods (RF, simple multi-layer perceptron)	$\bullet\ $ simple multilayer perceptrons or RFs often performed similarly or better compared with the tested deep learning models
Eckhart *et al*. (2024) [75]	$\bullet\ $ Four conventional ML methods (elastic net, RF, boosting trees, (deep) neural networks) $\bullet\ $ one state-of-the-art method (deep neural network) $\bullet\ $ nine-dimensional reduction techniques $\bullet\ $ 30 different feature numbers $\bullet\ $ two omics-types (expression, mutation)	$\bullet\ $ elastic net and ridge regression performed best $\bullet\ $ simple models with small feature numbers can outperform more complex models $\bullet\ $ the choice of ML algorithm and dimension reduction technique can substantially impact prediction performance $\bullet\ $ drug-sensitive samples are relatively poorly predicted

Especially the latter finding has implications for personalized treatment recommendation: it means that we have issues identifying successful treatments. While this problem is often overlooked, classification approaches such as LOBICO [19] and MERIDA [20] and regression approaches such as RWEN [36], and SAURON-RF [21] place particular emphasis on improving performance for drug-sensitive samples.

## Reliability

The evaluation of performance measures can indicate performance for future predictions, thereby creating trust in a model. However, it is merely one aspect in that regard. During the model deployment phase, i.e. when we apply a model to an unseen instance without a known response, we cannot evaluate a performance measure. Indeed, in real-world (healthcare) applications, the response is usually unknown, and we need an estimate of the extent to which we can trust a prediction, e.g. a probability estimate. In the ML literature, the degree of trust that we can have in a prediction for a single, previously unseen instance is referred to as reliability [69, 76, 77]. This definition serves as basis for the following discussion on reliability in this review paper.

One possibility to achieve reliability is via uncertainty quantification [78, 79]. Here, we are interested in estimating the uncertainty about the prediction for a specific instance, also called predictive uncertainty [52, 78]. Through uncertainty estimation, an ML model can be enabled to abstain from casting a prediction for a new instance if it is not certain enough, rendering the corresponding model more reliable.

Since the prediction for a new sample constitutes the end of an ML pipeline, the predictive uncertainty contains all uncertainties from data generation to the trained model. Two important types of uncertainty currently distinguished in the ML literature are called aleatoric and epistemic uncertainty [52, 79]. Aleatoric uncertainty denotes the data-inherent uncertainty caused by the randomness in the data-generating experiment, e.g. noise [52]. In supervised learning, the aleatoric uncertainty corresponds to the term $\epsilon $ that we introduced in Equation (1). Aleatoric uncertainty is irreducible, i.e. it cannot be eliminated by collecting more samples. In contrast, epistemic uncertainty arises from the lack of knowledge about the model that best approximates $f$ [52]. Thus, it can potentially be reduced by gathering more samples, which is why it is called reducible [52]. Hüllermeier and Waegeman [52] partition epistemic uncertainty into two sub-types called model and approximation uncertainty. The former refers to the uncertainty introduced by the choice of our hypothesis space $\mathscr{H}$, while the latter denotes the uncertainty in the estimation of the model parameters, which depends on the used training data. Thus, it is also referred to as estimation or parametric uncertainty in the literature instead [79]. There exists a direct connection between these uncertainty definitions and the bias-variance tradeoff from statistics [79]. The bias-variance decomposition of the expected squared loss of an ML model can be written as [80]


(4) \begin{align*}& E[(y- \hat{f}(x))^{2}] = \textrm{Var}(\hat{f}(x)) + [\textrm{Bias}(\hat{f}(x))]^{2} + \textrm{Var}(\epsilon).\end{align*}

While we cannot reduce $\textrm{Var}(\epsilon )$, we need to minimize the bias and variance to minimize the expected loss. The bias corresponds to the aforementioned model uncertainty and the variance corresponds to the approximation uncertainty.

Even though reliable predictions are highly desirable in a medical application, we found that only two papers of the current drug sensitivity prediction literature take this into account [29, 30] (cf. Table 1).

Fang et al. [29] use a quantile regression random forest (RF) to predict intervals instead of single drug response values. Clearly, intervals are more likely to hit the true value than point predictions. Moreover, the interval length provides information on uncertainty: given a specific quantile regression forest, a shorter interval corresponds to a higher certainty of the prediction. However, this approach is not sufficient to quantify reliability since no specific confidence level is guaranteed. Fang *et al.* [29] also assess the sharpness (i.e. the variability of predictions or predicted probability distributions): for a given sample and drug, they generate predictions for different quantiles and compute their variance. Fang *et al.* [29] assume that a smaller variance corresponds to a more stable prediction. They can leverage this assumption to compare cases where point predictions of a specific quantile are similar (e.g. one drug treatment versus another drug treatment for the same sample) using the Levene test [81]. The prediction is more stable if one case has a significantly lower variance.

Recently, we proposed a conformal prediction (CP) framework to ensure the reliability of predictions [30]. Our CP framework is not only applicable to regression but also classification, and we exemplified its capability using our previously published simultaneous regression and classification approach SAURON-RF [21]. CP represents a mathematically rigorous uncertainty quantification approach, which can generally be applied to any ML model that delivers a notion of (un)certainty [82]. This notion of (un)certainty may correspond to predicted class probabilities for classification, or estimated quantiles for regression. Based on this notion and a given user-specified maximum error level $\alpha \in [0,1]$, CP derives reliable intervals (regression) or sets (classification). These intervals and sets are guaranteed to contain the true value with a certainty of at least $1-\alpha $, known as the CP certainty guarantee [82]. For regression, we also employed quantile regression. Unlike the intervals by Fang *et al.* [29], our intervals are modified such that they fulfill the CP certainty guarantee. For classification, we use the predicted class probabilities to construct reliable sets using three different approaches.

## Interpretability

As outlined in the introductory section of this review, performance evaluation suffers from two major drawbacks. We addressed the first drawback, i.e. the inability to quantify performance for an unseen sample, in the previous section. In the following text, we discuss interpretability, a solution to the second drawback, i.e. the inability to provide explanations or justifications for the ML system.

**Figure 2:**
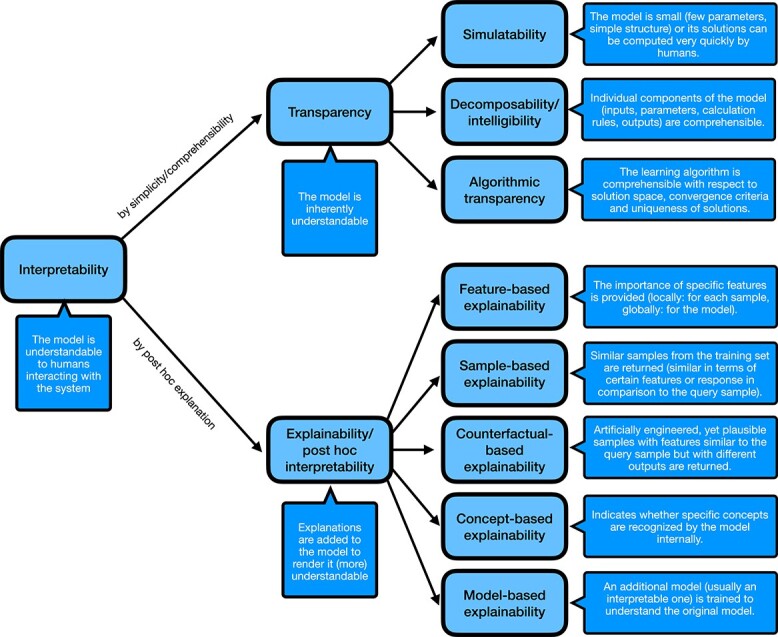
**Taxonomy of interpretability in ML;** this figure depicts a taxonomy of interpretability in ML that we derived from works by Lipton [47], Biran and Cotton [85], and Imrie, Davis, and van der Schaar [84]; the speech bubbles provide brief, intuitive descriptions of the technical terms; note that a specific ML model may possess properties from all of the categories in the bottom layer.

Intuitively, interpretability can be defined as the extent to which humans interacting with an ML system can understand its decisions and the underlying model [83–85]. While all of us have some intuition and preconceptions about what should constitute interpretability, from a mathematical perspective, no universally agreed approach to achieve or merely assess it exists [46, 86]. A system allowing for the distinction of different types of interpretability is absent [28]. Thus, we reviewed the ML literature to derive a taxonomy able to capture all prevailing notions of interpretability (cf. Fig. 2). Our taxonomy is mainly based on the works by Lipton [47], Biran and Cotton [85], and Imrie, Davis, and van der Schaar [84]. Generally, we distinguish two types of model interpretability: model-inherent interpretability, which is also known as model transparency [47], and interpretability generated from post hoc explanations denoted as explainability [84]. These two types can be further subdivided, and a specific ML model can possess characteristics from several subtypes. Notably, a model can be both transparent and explainable.

The subtypes of model transparency include the following:

- **Simulatability** [47] is the simplicity of a model in its entirety. Lipton distinguishes between two forms of simulatability: simulatability because of model size (e.g. a low number of parameters) and simulatability because of a low time requirement to perform manual model inference.
- **Decomposability/Intelligibility** [47, 87] is the comprehensibility of individual model components (inputs, parameters, calculation rules, outputs) and their correspondence to real-world phenomena (e.g. genes, biological pathways, cell lines). Ideally, each component of a model would be individually interpretable to fulfill this notion of interpretability as defined by Lipton [47] and Lou *et al.* [87]. However, often only parts of a model are understandable. Moreover, even if all parts are understandable, their interplay may preclude interpretation of the entire model.
- **Algorithmic transparency** [47] is the comprehensibility of the learning algorithm including the solution space, convergence criteria, or uniqueness of the solutions. Consider linear models: the shape of the error function is known, and training converges to a unique solution. In contrast, deep learning methods provide none of these benefits.

To maximize trust in an ML model, some may argue that interpretability in this strict sense has to be achieved. However, transparent models can be too simple to represent real-world phenomena, causing them to suffer from high bias (cf. Section 4 (Reliability)). Consequently, commonly applied ML algorithms, e.g. neural networks, are of a black-box nature instead. Similarly, human decision-making is not transparent. Indeed, what we do have are explanations and justifications that humans provide, rendering them similar to black-box ML models augmented with post hoc explanations. While post hoc explanations are typically employed when models are not transparent, even inherently transparent models can benefit from them.

Following the work by Imrie, Davis, and van der Schaar [84], we divide post hoc explanations into five categories:

- **Feature-based explainability** refers to the importance of features, either locally for specific samples or globally across the model. Arguably, feature-based explainability is the most widely applied explainability method: for instance, most implementations of tree-based methods supply users with the built-in functionality to calculate features importances. Here, each input feature is attributed an importance based on the quality of the decision splits in terms of error reduction and position of the feature in the tree(s).
- **Sample-based explainability** aims to identify samples from the training data that the model views as being similar to a given sample. Similarity can, for instance, be based on the features or the predicted response of the given sample. At first, the returned set of training samples does not explain why the prediction is cast. The hope is that the user has domain knowledge for the samples in the training data, which helps to interpret the prediction for the given sample. By closer inspection of the properties of the training samples, putatively interesting features could be identified. To this end, it might be beneficial to employ feature-based explainability methods.
- **Counterfactual-based explainability** refers to the generation of artificially engineered, yet plausible samples that are similar to the query sample but generate a different output. Typically, the idea is to modify as few features of the query sample as possible to identify features that might be linked to changes in the response.
- **Concept-based explainability** refers to the examination whether specific concepts (e.g. patterns in an image) are recognized by the model. Usually, methods that implement concept-based explainability compare a set of samples with the concept to a set of samples without the concept and assess whether internal representations of both sets in the model differ. Such representations could include the activation of certain nodes in a neural network or traces that samples take through individual trees in an RF.
- **Model-based explainability** denotes the generation of a second, more transparent model trained on the original model. The goal of the second model would be to elucidate the decision process of the original model. Thus, rule-based models lend themselves well to be used as second model.

One interpretability type may not suffice to answer all questions that the different stakeholders, e.g. patients, clinicians, and statisticians, may have. Thus, incorporating several interpretability types into one model is often advisable: feature-based explainability may be most suitable to represent tumor characteristics that drive the prediction. Counterfactual explanations may be most suited to derive recommendations for therapeutic interventions that may change the outcome, e.g. lifestyle changes (dieting, smoking, drinking habits, etc.). Concept-based methods are an important cornerstone of model validity; they should indicate whether samples with known biomarkers of drug response are correctly treated by the model, i.e. the model acts as expected. If not, the model might not be informative or could have discovered novel associations. Imrie, Davis, and van der Schaar provide a comprehensive overview on questions of different stakeholders and explainability types helping to answer them [84].

**Figure 3:**
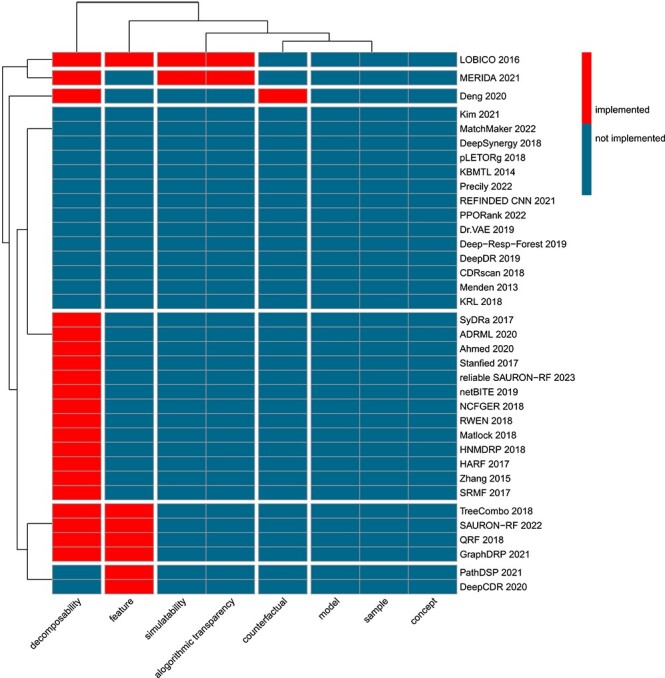
**Hierarchical clustering of interpretability types;** this figure depicts a clustering of the 36 investigated approaches for drug sensitivity prediction based on our taxonomy (cf. Fig. 2).

For each drug sensitivity prediction approach, we checked which types of interpretability have been implemented (Table 1). Figure 3 shows a hierarchical clustering of these data. Contrary to what we would wish for, several approaches consider none of the discussed interpretability types and most other approaches focus on only a few. We find that the number of papers per interpretability type varies. In the following text, we discuss the interpretability types from least to most frequent by reviewing all associated approaches. Note that neither model- nor sample-based explainability was implemented by any of the 36 approaches, which is why we exclude them from the following discussion. There were also no approaches that presented or utilized automatized evaluation methods for the detection of concepts. However, since the verification of successful detection of concepts occupies a special position in natural sciences, we will briefly discuss partial realizations of this type of explainability.

### Concept-based explainability

Virtually all of the analyzed methods provide some analysis on whether the model and its results align with commonly known phenomena of drug response: they test whether samples with known biomarkers of drug response are correctly treated by the model. Examples of such biomarkers include mutations, gene expression patterns, or pathway activities. For example, mutations in BRAF are linked to increased sensitivity to MEK inhibitors [88], which Menden *et al.* [61], Zhang *et al.* [31], Liu *et al.* [37], and Chawla *et al.* [64] investigated. This type of analysis is already moving in the direction of concept-based explainability. However, there are two noteworthy differences: First, the analyses were not automated. Second, the analyses did not investigate the internal state of the model but only its output. For neural networks, there exist automated methods such as TCAV [89] and CAR [90] that allow for comparing samples with and without a particular concept in the latent space of the neural network.

### Counterfactual-based explainability

Deng *et al.* [39] trained a neural network. As input features, they consider gene expression and drug–protein interaction scores. Their network contains a pathway layer, where each node corresponds to a biological pathway, and the output of each node can be interpreted as a measure of pathway activity. By generating artificial inputs (setting drug–protein interaction scores of all targets to zero), Deng *et al.* [39] could compare the calculated pathway activities with and without drug treatment. They found that drugs targeting a certain pathway reduce the activity of the target pathway for the original (with drug treatment) samples.

### Algorithmic transparency and simulatability

LOBICO [19] and MERIDA [20] are both based on integer linear programming (ILP), which is concerned with optimizing a system of linear (in)equalities over a set of integer decision variables. There exist exact problem-solving algorithms for ILPs [91], e.g. branch-and-bound algorithms, cutting plane methods, and a combination of the two, branch-and-cut algorithms. Moreover, the solution space of ILP approaches is relatively well studied. These properties render ILP approaches algorithmically transparent. For LOBICO and MERIDA, the employed (in)equalities model Boolean rules specifying sensitivity or resistance to a particular drug, and the corresponding ILP solution represents the logic rule that best explains the observed drug responses of the training samples. Since the derived rules are relatively small in terms of the considered input features, a human could easily classify novel samples, i.e. the models are simulatable.

### Feature-based explainability

Both SAURON-RF [21] and QRF [29] model drug response prediction with regression RFs. Conventional implementations of RFs augment them with post hoc explanations in the form of feature importances [92, 93]: one possibility is to quantify how much the feature improves prediction error during splitting (impurity-based); another possibility is to shuffle the feature values across the samples and assess the resulting degradation of model performance (permutation-based). These methods were also employed by SAURON-RF and QRF, respectively.

PathDSP by Tang and Gottlieb [42] and TreeCombo by Janizek et al. [24] rely on SHAP (SHapley Additive exPlanations) values by Lundberg and Lee [94], an extension of Shapley values [95] introduced by Lloyd Shapley in 1953 to attribute the contribution of a player to a game result. In ML, Shapley and SHAP values are commonly applied to estimate the contribution of a feature to a prediction. They can be used to obtain both sample- and model-specific feature importances.

Knijnenburg *et al.* [19] compute feature importances that for each feature reflect the change in error between the original model and the model without the feature. This method is derived from a variable activity measure used in Boolean networks [96, 97].

For GraphDRP Nguyen *et al.* [43] investigated feature attributions by inspection of saliency maps [98]. Saliency maps are typically employed in image processing and computer vision applications to visualize the most relevant pixels of an image [99].

Lastly, in DeepCDR, Liu *et al.* [44] compute feature importances for an individual sample in their deep neural network using the gradient of the predicted response with respect to each feature.

### Decomposability

Decomposability was by far the most often employed interpretability type for drug sensitivity prediction in cancer (20 out of 36 investigated models).

Partially, this is the direct result of the fact that several approaches use inherently interpretable model types, i.e. ILP [19, 20], RFs [21, 25, 29, 30, 33, 35, 38], boosting trees [24], and elastic net [36]. An integer linear program can be decomposed into individual constraints. For LOBICO [19] and MERIDA [20], these constraints directly correspond to the rules for drug sensitivity or resistance. Similarly, an RF can be divided into single trees that each generate straightforward if-then-else rules for decision-making when tracing a route from the root to a leaf. Lastly, elastic nets are linear models where each prediction consists of a linear combination of features multiplied by their coefficients, which can be interpreted as feature attributions.

A substantial proportion of the remaining decomposable approaches exploits the similarity between cell lines, drugs, or other biological entities, often combined with modeling interactions between entities [31, 32, 34, 37, 40, 41]. Many approaches represent these similarities as graphs where nodes correspond to the different biological entities and edges between nodes are weighted by the similarity between the corresponding entities. While such graphs can be rather large, the individual components can easily be understood by a human, rendering the models decomposable. The standard procedure of these methods entails the computation of cell line similarity on omics-profiles and drug similarity on molecular properties or fingerprints. Depending on whether the considered data types are continuous or discrete, different similarity and distance measures are employed. Usually, Pearson correlation (e.g. [31, 32, 34, 37, 40, 41]) is used for continuous data and the Jaccard similarity coefficient (e.g. [32, 37, 41]) for binary data.

In the last group of decomposable approaches, other types of graphs are used to render the models decomposable. Nguyen *et al.* [43] model the molecular structure of a drug as a graph, where nodes are atoms and edges represent bonds between atoms. In contrast, Stanfield *et al.* [45] use graphs to depict protein–protein interactions, the presence of mutations in cell lines, and drug sensitivity. Lastly, Deng *et al.* [39] incorporate a pathway layer into their neural network, which can be interpreted as a graph that connects all available genes to their respective pathways.

## Perspectives

Overall, our review of the 36 articles revealed that hardly any reliability research has been carried out over the last decade: only two articles take this into account. Interpretability has been considered an essential concept in 22 articles. While these 22 articles considered different connotations of interpretability, they did not provide a definition of the type of interpretability they investigated. In the ML literature, interpretability is known to be an elusive concept that is difficult to formalize, leading to ambiguities and misconceptions [46, 47]. To address this issue, we proposed a general taxonomy for interpretability using the existing body of knowledge in ML and categorized the drug sensitivity prediction approaches accordingly. Based on our findings, we will discuss promising future research directions for trustworthiness in ML-based drug sensitivity prediction in the following text. We start with reliability, then cover interpretability, and finally highlight other important aspects of trustworthiness, e.g. data security, safety, and privacy.

### How to achieve reliability

Only a few approaches to achieve reliability have been investigated for drug sensitivity prediction in cancer. Common to all of the mentioned ML methods and reliability estimation approaches described up to this point is that they rest on the idea that the data used to train and test the ML model were drawn iid. Given this assumption, CP as, for example, applied by Lenhof *et al.* [30], guarantees the validity of the intervals or sets produced from a model [82, 100]. Yet, in real-world application scenarios, this assumption is unlikely to hold. Among others, there can be shifts in the input (feature) space (called covariate shifts [101, 102]), the conditional probability distribution of the response on the features (called concept shifts [101]), and the response labels (called label or semantic shifts [102]), i.e. by introduction or alteration of response labels. Consequently, additional countermeasures are required to achieve reliability under these circumstances. In particular, we expect that human tumor data deviate from our model system-based training data. If we know about the shift, we may mitigate its effects, e.g. using a modified CP procedure [82]. In our application case, we may, for example, be able to quantify a covariate shift by comparing molecular tumor data with the cell line profiles. In many cases, however, quantifying shifts will hardly be feasible. For instance, *in vitro* and *in vivo* cellular mechanisms of drug responses likely differ, introducing a concept shift. Yet, we cannot detect or quantify it without sufficient amounts of tumor omics and drug response data. Thus, we would prefer models that generalize well to out-of-distribution data, e.g. as described by Liu *et al.* [101]. Note that semantic shifts as described by [102] are unlikely to occur in drug sensitivity prediction: Our drug response data entail the complete sensitivity scale from highly resistant to highly sensitive. We can partition the scale into arbitrarily fine-grained classes (e.g. sensitive, ambiguous, and resistant samples) but any definition will always cover the scale in its entirety. Consequently, an unseen sample cannot belong to any new class.

### How to achieve interpretability

Most of the investigated publications on drug sensitivity prediction acknowledge the importance of having interpretable ML models and attempt to incorporate some type of interpretability into their models. By far, the most frequently and often the solely used interpretability type was decomposability, which refers to the understandability of single components of a model. However, the intelligibility of each component does not imply the interpretability of the entire model: there can be many components involved in a complex interplay, precluding interpretations, explanations, or justifications of model predictions. Moreover, in Section 5 (Interpretability), we discussed that it is crucial to consider several interpretability types at once to address the diverse set of questions that arise during medical decision support and to render models as interpretable as possible. Indeed, many of the 36 investigated approaches need additional explainability layers to provide meaningful explanations for model predictions. Here, it would be particularly interesting to investigate the types of interpretability that have not or only rarely been considered: concept-based, sample-based, model-based, and counterfactual-based explainability. For all these types of explainability, Imrie *et al.* [84] summarize methods that realize them. While some methods can sit on top of any ML model, others are limited to certain models, e.g. neural networks. On the other hand, the question arises to what extent the subsequent addition of explainability to complex models is required given the current data scarcity. There is increasing evidence that relatively simple (transparent) methods currently suffice to predict anti-cancer drug response (cf. [19, 20, 74]) and that the apparent outperformance of complex deep neural networks might be linked to data leakage [103] or technical artifacts instead [74]. Like Rudin [86], we believe simple models should be preferred as long as complex ones do not significantly outperform them. Moreover, these simple models can also be augmented by explainability methods.

### Combining reliability and interpretability

In principle, reliability and interpretability are independent concepts, i.e. one can be achieved without the other. To become trustworthy, however, we want our model to fulfill not only one of the trustworthiness-related properties but all of them simultaneously, e.g. in our case, models should be both reliable and interpretable. From our analysis, it becomes apparent that only two approaches (Fang *et al.* [29] and Lenhof *et al.* [30]) consider reliability and interpretability at once. Yet, neither of the approaches addresses the question of how to interweave the two concepts. For instance, we could pursue the goal of assessing the reliability of the explanations of an ML model. Likewise, we could derive explanations for the confidence that we have in predictions.

### Tailoring models to real-world applications to increase trustworthiness

While numerous ML algorithms for drug sensitivity prediction exist, it is crucial for these models to effectively address real-world issues to be truly valuable in decision support systems. In this context, we have focused on rendering the models more reliable and interpretable. However, further aspects of model design need to be considered in that respect. For example, most of the approaches we discussed are concerned with optimizing the prediction of some drug response measure. However, the true aim of a decision support system would be to prioritize drugs, i.e. to provide a list of recommendable drugs sorted by their efficiency. Currently, only a few approaches cover this overarching question (PPOrank [65], KRL [59], pLETORg [55], reliable SAURON-RF [30]). For this task, accurate predictions for the drug-sensitive samples are paramount. However, as described in Section 3 (Performance), this is an unresolved issue mainly caused by the current data scarcity. Given this scarcity—especially for drug-sensitive samples—surprisingly little attention has been paid to the potential of combining data from different model systems (such as cell lines, patient-derived xenografts, and organoids) and even publicly available tumor data to increase the robustness of the predictions. While data scarcity is already an issue for training models to predict responses to single drugs, training synergy prediction models is even more challenging because of the exponentially growing number of putative drug combinations severely limiting the number of feasible lab experiments. Consequently, combining data from various sources, including monotherapy and drug interaction data, is crucial to estimating the effect of two or more simultaneous treatments and potential polypharmacy side effects arising from drug combinations [104–106]. On top of that, combining data from heterogeneous sources is also essential for clinical applications since clinics often have private data that they would like to combine with publicly available sources. Methods such as transfer-learning and meta-learning could be leveraged to accomplish this objective. We refer the interested reader to comprehensive surveys by Zhuang *et al.* [107] and Vanschoren [108] for further information.

Another relevant question regarding the usefulness of models in the clinic is how the results of (bulk sequencing from) model systems can be transferred to single-cell sequencing data of patients, where the heterogeneity of a tumor is represented by sequencing results from different tumor clones, each of which may have a unique drug response [109].

In this article, we discuss the use of methods for drug sensitivity (and synergy) prediction in the context of decision support systems. However, these methods and all trustworthiness-related concepts presented in this paper can also be valuable tools in the drug discovery pipeline, where the estimation of drug sensitivity, synergy, toxicology, and side effects is an important goal as well.

### Other important aspects of trustworthiness

This review focused on reliability and interpretability as essential factors of trustworthiness particularly crucial for designing an ML method. When embedding such an ML method into a decision-support framework, other factors of trustworthiness also play at least as important a role [110]. For instance, the following factors may not be relevant for the development of ML models on publicly available model-system data, but are crucial for the deployment of ML models in decision support systems working with patient data:

- privacy mechanisms protecting the used data,
- security mechanisms protecting the system against external threats and intentional misuse,
- safety mechanisms protecting the system against accidental misuse,
- bias-awareness and fairness of the system, protecting against harm caused by the usage of data considered sensitive, e.g. ethnicity or gender.

Qayyum *et al.* [111] provide an overview of privacy and security mechanisms for clinical treatment recommendation using ML models. Another crucial factor for real-world deployment of ML models in decision support systems is the human interaction with this system: If the system is difficult to use, and information (e.g. treatment recommendations and justifications thereof) is not presented clearly and intuitively, this can significantly reduce the user’s trust in a system. Consequently, user-friendliness should be a primary concern when developing decision support systems. They should be tailored to specific user groups, including medical doctors, bioinformaticians, or patients. Usability studies are thus required to determine factors that contribute to or impede system usability. In order to meet all the requirements of a trustworthy decision support system, interdisciplinary cooperation is thus necessary. This should not only include researchers and professionals from the life sciences such as medical doctors, biologists, pharmacists, and bioinformaticians, but also security researchers, psychologists, and UI/UX designers.

**Key Points**

- We present a comprehensive review of ML-based anti-cancer drug sensitivity approaches encompassing 36 articles published during the previous decade.
- We place the approaches in the four major ML realms and analyze to what extent the trustworthiness-related properties *reliability* and *interpretability* have been considered for model development.
- On the one hand, our analysis reveals that reliability has hardly been considered during model development despite being of utmost importance for deploying ML systems in practice.
- On the other hand, interpretability has often been considered. However, the concept is used rather intuitively, lacking clear definitions.
- To solve the latter problem, we propose a sound and easily extensible taxonomy of interpretability that will serve as a reference for the development of future methods.

## Data Availability

Not applicable.
